# Enhancing abdominal muscle thickness and sitting ability in spastic hemiplegic children via whole-body vibration: a randomized controlled trial evaluated by ultrasonography

**DOI:** 10.11604/pamj.2026.54.24.48867

**Published:** 2026-06-02

**Authors:** Mamdouh Gabr Haggag, Daniel Kayode Adejumo, Neda Mohamed Yousef, Mohamed Zakaria El-Sayed, Praveen Kumar, Mohammad Rawashdeh, Magdi Ali Abdou Gouda

**Affiliations:** 1 Department of Physiotherapy, College of Health Sciences, Gulf Medical University, Ajman, United Arab Emirates,; 2 Pediatric Physical Therapy Department, Faculty of Physical Therapy, Cairo University, Cairo, Egypt,; 3 Rehabilitation and Diagnostic Sciences Department, College of Medical and Health Sciences, Liwa University, Abu Dhabi, United Arab Emirates,; 4 Medical Imaging Sciences, College of Health Sciences, Gulf Medical University, Ajman, United Arab Emirates,; 5 Faculty of Health Sciences, Jordan University of Sciences and Technology, Irbid, Jordan

**Keywords:** Cerebral palsy, whole-body vibration, abdominal muscles, ultrasonography, sitting ability, trunk control

## Abstract

**Introduction:**

cerebral palsy (CP) affects trunk control and motor development. Weak abdominal muscles and poor sitting posture are common in spastic hemiplegic CP. Core muscle strength is essential for functional mobility. Whole-body vibration (WBV) may enhance muscle thickness and postural control. This study examined the effect of WBV on abdominal muscle thickness, sitting ability, and gross motor function in children with spastic hemiplegic CP.

**Methods:**

a randomized controlled trial design was used. Thirty children with spastic hemiplegic CP (ages 5-10 years) were randomly assigned to two groups. Group A received conventional physical therapy. Group B received the same program, plus 10 minutes of WBV, three times per week for 12 weeks. Outcomes included abdominal muscle thickness via ultrasonography and gross motor function via Gross Motor Function Measure-88 (GMFM-88). Pre- and post-intervention measures were compared within and between groups using paired and independent t-tests. Significance was set at p<0.05.

**Results:**

no significant baseline differences were found between groups. After the intervention, both groups showed improvement. However, group B showed greater increases in abdominal muscle thickness (Rectus Abdominis (RA): 6.06%, Internal Oblique (IO): 8.89%, External Oblique (EO): 5.88%, Transverse Abdominis (TA): 9.68%, and GMFM-88 scores). Group B also demonstrated significant improvement in sitting balance.

**Conclusion:**

whole-body vibration (WBV) can be a practical tool to strengthen core muscles and improve trunk control in children with spastic hemiplegic CP. Physiotherapists may consider integrating WBV into conventional therapy to improve functional outcomes. Further studies should explore long-term effects and optimal WBV parameters.

## Introduction

One of the most prevalent factors leading to motor dysfunction in children is CP. This condition significantly affects developmental milestones, including sitting, standing, functional abilities, and postural control [1,2]. Skeletal muscles exhibit remarkable adaptability and plasticity, allowing them to adjust normally or abnormally in length and tension in response to any type of loading [1]. Postural stability, particularly in sitting, is a crucial milestone required for lumbar spine integrity, and if a child cannot maintain an erect posture in sitting, it indicates incoordination in the trunk, pelvis, lower limbs, and buttocks musculature [3]. Additionally, core muscles such as Internal Oblique (IO), External Oblique (EO), Rectus Abdominis (RA), and Transversus Abdominis (TA) are vital for trunk stabilization and maintaining lumbar spine integrity [4,5].

Given the association of core muscles with truncal control, abdominal muscle thickness and size become crucial for trunk and balance control [6]. For this purpose, a non-invasive method such as ultrasonography can be used to assess and provide insights into the muscle architecture of children with CP [7,8], and it can be applied during both muscle activation and the resting state [9].

During rehabilitation sessions, physical therapists leverage the plasticity phenomenon and emphasize training abdominal muscles to enhance motor function and postural control [10,11]. As mentioned earlier, children with CP have poor trunk control, leading to excessive anterior pelvic tilting [10,12]. This mechanism stretches the abdominal muscles, particularly the RA and TA, thereby impairing their ability to maintain a neutral pelvis [13]. Therefore, strengthening them is highly recommended to develop trunk control, promote healthy bone and musculature growth, and improve motor function in CP [10-13].

Numerous studies have explored the effectiveness of WBV in strengthening core muscles and improving the ability to walk in children with CP [14,15]. Studies have proven that WBV improves bone mineral density [16], strength, and functional performance such as walking, as well as assists in controlling spasticity and improving gross motor function, leading to an overall improvement of mobility and function in these children [17-19]. This is why WBV has garnered attention as a versatile strategy for enhancing muscle strength across various practical domains [20,21].

Our study aims to investigate the impact of WBV on abdominal muscle thickness, sitting ability, and trunk control in children with CP and spastic hemiplegia. Our hypothesis posits that WBV will significantly enhance sitting ability and increase abdominal muscle size, resulting in improvement in postural control and day-to-day activities.

## Methods

**Study design:** this study employed a two-arm, parallel-group randomized controlled trial (RCT) comparing a conventional physiotherapy program (control) with the same program supplemented by WBV (intervention). Participants were randomly allocated to groups, with allocation concealment and blinded outcome assessment to minimize bias.

**Study setting and population:** this study was conducted at Thumbay Physical Therapy and Rehabilitation Hospital, a specialized rehabilitation facility affiliated with Gulf Medical University, located in Ajman, United Arab Emirates. The hospital is a referral center that provides comprehensive physiotherapy and rehabilitation services for pediatric and adult populations with neurological and musculoskeletal conditions. Data collection and intervention procedures took place between April 2024 and May 2025. The study population consisted of children diagnosed with unilateral spastic CP who were receiving care at the hospital during the study period. A total of 30 participants were enrolled and randomly allocated to two groups: 15 to the control group and 15 to the intervention group. Participants were between 5 and 10 years of age, representing both sexes. Recruitment was carried out through referrals from the hospital's pediatric neurology and rehabilitation clinics, and a physiotherapist screened all eligible participants before enrollment.

**Inclusion criteria:** children classified as GMFM-88 levels I-IV and presenting with mild to slightly more severe spasticity, corresponding to grades 1 or 1+ on the modified Ashworth Scale [22-24].

**Exclusion criteria included:** (i) undergoing orthopedic or neurosurgical procedures intended to reduce spasticity within the prior 6 months; (ii) receiving botulinum toxin injections for spasticity management within the prior 6 months; (iii) recent orthopedic casting within the past 6 months; and (iv) having a body mass index (BMI) ≥24.9 kg/m^2^, as excess adiposity may affect ultrasonographic measurement accuracy.

**Variables and outcomes:** the primary outcomes were thicknesses (mm) of the RA, IO, EO, and TA muscles measured by ultrasonography. The secondary outcome was gross motor function assessed with GMFM-88. Additional quantitative descriptors included age (years) and GMFM-88 scores (%). Categorical descriptors included sex, degree of spasticity, and side of hemiplegia.

### Data sources and measurement

#### Data collection

**Ultrasonography:** muscle thickness was acquired with a 7.5 MHz linear array probe. For EO, IO, and TA, the transducer was placed on the right abdomen at the upper end of the umbilicus; TA was measured 2-5 cm lateral to the umbilicus with the site marked to ensure repeatability. Still images were extracted from 5-second ultrasound videos, and thickness was defined as the perpendicular distance between superficial and deep aponeuroses. Procedures, positioning, and acquisition parameters were standardized across participants, and all scans were performed by the same experienced therapist to limit inter-rater variability [25].

**Gross motor function:** Gross Motor Function Measure-88 (GMFM-88) (88 items across five domains: lying and rolling (dimension A); sitting (dimension B); crawling and kneeling (dimension C); standing (dimension D); walking, running, and jumping (dimension E)) was administered according to validated guidance [26]. Sitting ability was specifically evaluated using dimension B (sitting) of the GMFM-88, which assesses the postural control and the functional performance of the children in the sitting position. Only dimension B scores were analyzed for the purpose of this study. Outcome assessors were blinded to group assignments.

**Randomization, allocation concealment, and blinding:** a computer-generated randomization sequence was created using SPSS software to assign participants in a 1: 1 ratio to either group A or group B. Allocation concealment was ensured using sequentially numbered, opaque, sealed envelopes prepared by an independent researcher who was not involved in participant recruitment or assessment. After baseline assessment, each participant´s group assignment was revealed by opening the next envelope in sequence. Outcome assessors were blinded to group allocation throughout the study. All ultrasound measurements were performed by a single trained therapist following a standardized protocol. The radiologist performing the ultrasonographic measurements and the assessor administering the GMFM-88 were blinded to group allocation throughout the study. The treating therapists were not blinded due to the nature of the intervention.

### Interventions

**Control (conventional physiotherapy):** participants in the control group received a standardized conventional physiotherapy program three times per week for 12 weeks, with each session lasting approximately 60 minutes. The program focused on improving trunk control (trunk flexion, extension, side flexion, and rotation), postural stability in the sitting position, balance, and lower limb flexibility. Exercises included seated trunk stabilization activities on a Swedish ball emphasizing anterior-posterior and lateral weight shifting; facilitated righting and equilibrium reactions of head and trunk in sitting position based on neurodevelopmental treatment principles; sitting on the spongy roll like horseback riding to ensure separation between the lower limb to enhance the trunk stability while the child is doing activities with his hand like reaching and grasping toys located in front of him; balance-board training to enhance dynamic postural control; and functional reaching tasks in seated and standing positions to promote core muscle activation. Changing position exercises from supine to sitting and from prone to sitting to facilitate the raising mechanism. Postural adjustment exercises, as the child tries to throw/catch a small Swedish ball from a different direction while he is sitting on the spongy roll or the Swedish ball, to ensure his capability to adjust the trunk posture during throw/catch movement. Stretching exercises targeting the hamstring, iliopsoas, and calf muscles were performed at the end of each session, with each stretch held for 20-30 seconds and repeated three times. Exercise intensity and task difficulty were progressively adjusted according to each child´s functional ability to maintain engagement and ensure safe challenge throughout the intervention period.

**Intervention (conventional physiotherapy + WBV):** in addition to the conventional program above, participants received WBV for 10 minutes per session (30 Hz frequency; 2 mm amplitude): 5 minutes in a squat position, 1-minute rest, and 5 minutes standing. Any discomfort or distress during WBV was monitored and documented.

**Sample size:** sample size was determined using G*Power software (version 3.1.9.2). Based on pilot data assessing RA muscle thickness, a large effect size (Cohen´s d = 1.1) was assumed, with a significance level of α = 0.05 and statistical power of 80%. This calculation indicated that a minimum of 15 participants per group was required, resulting in a total sample size of 30 children. Post hoc power analysis confirmed high achieved power for the primary outcome measure. The effect size was selected based on previously reported large improvements in functional strength and normalization of muscle tone before functional mobility intervention in children with spastic CP following WBV interventions in pediatric neurological populations [17,27].

**Data analysis:** continuous variables (muscle thicknesses and GMFM-88 scores) were summarized as mean ± standard deviation; categorical variables (sex, spasticity grade, and side of hemiplegia) as frequencies and percentages. Between-group comparisons for categorical variables used chi-square tests. For continuous variables, independent-samples t-tests compared groups at baseline and post-intervention, and paired t-tests assessed within-group change from pre- to post-intervention. A two-sided 95% confidence interval was applied, and significance was set at p < 0.05. Analyses were conducted in SPSS v19.

Several comparisons were performed across different muscle groups and functional results. Due to the exploratory aspect of secondary outcomes and the limited sample size, no formal adjustments for multiple testing were applied to minimize the risk of type II error and the possibility of masking the clinically relevant effects. Nevertheless, results must be viewed carefully, considering the possibility of increased type I errors.

**Quality assurance and bias control:** methodological safeguards included centralized computer-generated randomization, sealed-envelope allocation, blinding of outcome assessors, standardized positioning and scanning procedures, and use of a single experienced sonographer. Participant adherence was tracked, and deviations from protocol were recorded.

**Ethical considerations:** the trial adhered to the Declaration of Helsinki. Written informed consent was obtained from participants' legal guardians prior to enrollment after full disclosure of study aims and procedures. Ethical approval was granted by the Ethics Committee of Gulf Medical University (IRBeCOHSeSTD-122-APR-2024).

## Results

**Participant flow and demographics:** participant flow is presented in the CONSORT diagram (Figure 1). Thirty children with spastic hemiplegic CP were assessed and randomly assigned to two groups: fifteen received conventional physiotherapy (group A) and fifteen received conventional physiotherapy with WBV (group B). All participants completed the intervention and were included in the final analysis. Baseline characteristics, summarized in Table 1, showed comparable mean age (group A: 5.60 ± 0.78 years; group B: 5.25 ± 0.80 years, T = -1.219, P = 0.116), sex distribution (group A: 7 males (46.7%), 8 females (53.3%); group B: 8 males (53.3%), 7 females (46.7%), Chi-square = 0.1333, P = 0.715), hemiplegic side (group A: 6 right (40%), 9 left (60%); group B: 8 right (53.3%), 7 left (46.7%), Chi-square = 0.536, P = 0.464), and spasticity grade (group A: 60% grade 1, 40% grade 1+; group B: 40% grade 1, 60% grade 1+, Chi-square = 1.2, P = 0.273). These results confirm that groups were equivalent at baseline.

**Figure 1:**
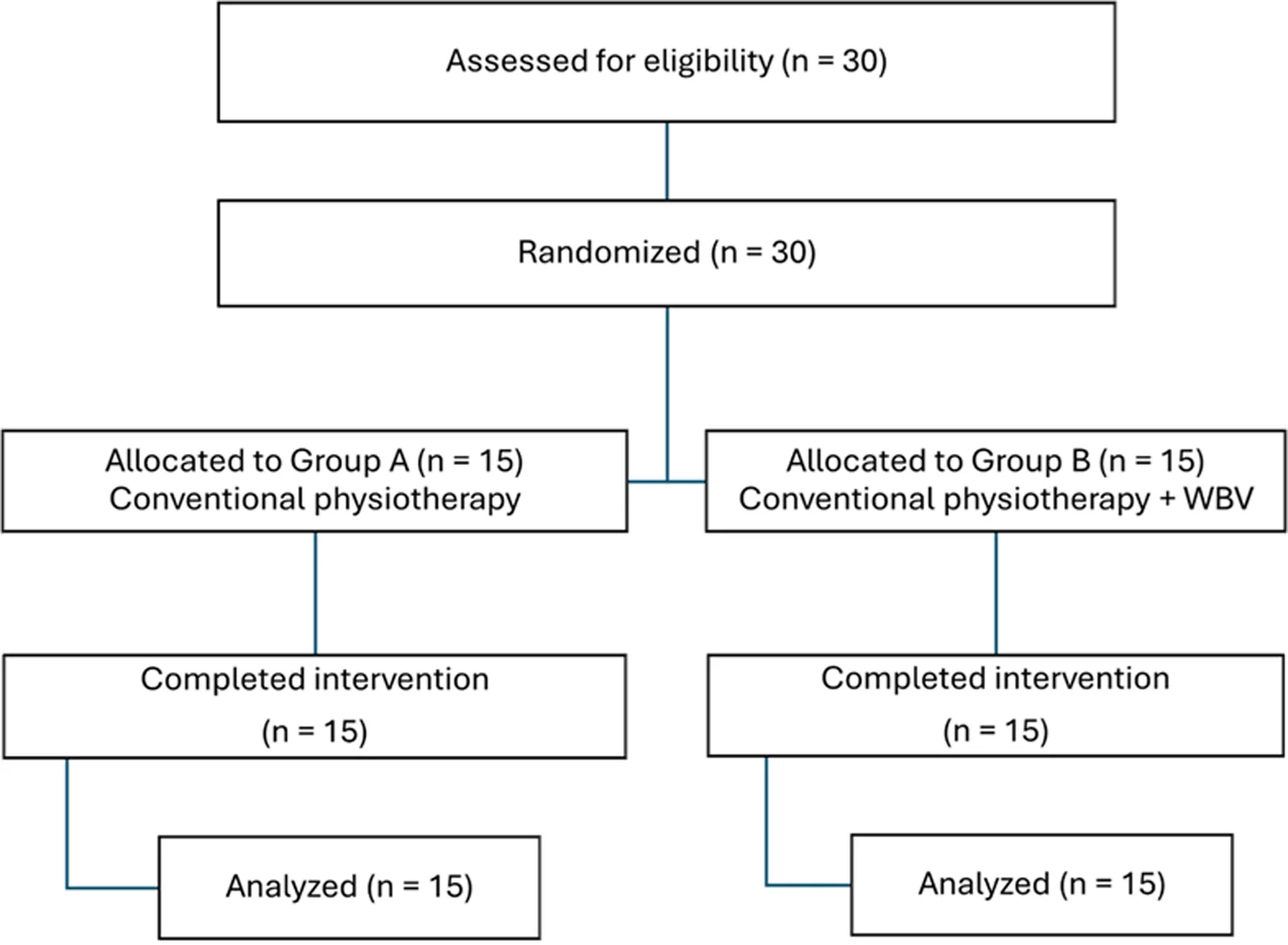
CONSORT flow diagram of participants through the study

**Table 1:** demographic characteristics of study participants, recruited from Thumbay Physical Therapy and Rehabilitation Hospital, United Arab Emirates, between April 2024 and March 2025 (N=30)

Variables		Group A	Group B	T Value	Chi-sq.	P value
Age (Y)	Mean ± SD	5.60 ± 0.78	5.25 ± 0.80	1.219	-	0.116 (NS)
Sex	Male	7 (46.7%)	8 (53.3%)	-	0.1333	0.715 (NS)
	Female	8 (53.3%)	7 (46.7%)			
Hemiplegic side	Right	6 (40%)	8 (53.3%)	-	0.536	0.464 (NS)
	Left	9 (60%)	7 (46.7%)			
Spasticity	Grade 1	9 (60%)	6 (40%)	-	1.2	0.273 (NS)
	Grade 1+	6 (40%)	9 (60%)			

Mean ± SD: mean ± standard deviation; NS: non-significant difference (P>0.05); Chi-sq.: Chi-square test; T value: unpaired t-test; P value: significance level at 0.05

**Impact of WBV on abdominal muscle thickness:** both groups showed improvements in abdominal muscle thickness after the intervention, with greater gains in group B (Table 2). RA thickness increased from 0.66 ± 0.02 to 0.70 ± 0.03 in group A (6.06%, t = 5.719, P < 0.01) and from 0.67 ± 0.03 to 0.76 ± 0.02 in group B (13.43%, t = 3.739, P < 0.001), with post-treatment comparison favoring group B (T = 6.445, P < 0.001). IO thickness increased from 0.45 ± 0.02 to 0.49 ± 0.02 in group A (8.89%, MD = 0.04, t = 11.835, P < 0.01) and from 0.46 ± 0.02 to 0.53 ± 0.03 in group B (15.22%, MD = 0.07, t = 5.449, P < 0.001), showing a significant between-group difference (T = 4.296, P < 0.001). EO increased from 0.34 ± 0.02 to 0.36 ± 0.03 in group A (5.88%, MD = 0.02, t = 11.826, P = 0.0202) and from 0.33 ± 0.04 to 0.40 ± 0.03 in group B (21.21%, MD = 0.07, t < 0.0001), with group B showing significantly greater improvement (T = 3.652, P = 0.0005). TA thickness increased from 0.31 ± 0.05 to 0.34 ± 0.04 in group A (9.68%, MD = 0.03, t = 11.832, P = 0.04) and from 0.29 ± 0.03 to 0.38 ± 0.02 in group B (31.03%, MD = 0.09, t < 0.0001), a significant post-treatment difference (T = 3.464, P < 0.001).

**Table 2:** baseline and post-treatment motor function and abdominal muscle thickness in children with cerebral palsy assigned to group A and group B, recruited from Thumbay Physical Therapy and Rehabilitation Hospital, United Arab Emirates, between April 2024 and March 2025 (N=30)

Variables		Mean ± SD		MD	%	Cohen	T value	P value	Sig.
		Before	After						
Rectus abdominis RA	Group A	0.66 ± 0.02	0.7 ± 0.03	0.04	6.06	2	5.719	< 0.01	HS
	Group B	0.67 ± 0.03	0.76 ± 0.02	0.09	13.43	3	3.739	< 0.001	HS
	T value	1.074	6.445	-	-	-	-	-	-
	Cohen	0.5	2	-	-	-	-	-	-
	P-value	0.146 (NS)	<0.001 (HS)	-	-	-	-	-	-
Internal oblique IO	Group A	0.45 ± 0.02	0.49 ± 0.02	0.04	8.89	2	11.835	< 0.01	HS
	Group B	0.46 ± 0.02	0.53 ± 0.03	0.07	15.22	3.5	5.449	< 0.001	HS
	T value	1.369	4.296	-	-	-	-	-	-
	Cohen	0.5	2	-	-	-	-	-	-
	P-value	0.0908 (NS)	< 0.001 (HS)	-	-	-	-	-	-
External oblique EO	Group A	0.34 ± 0.02	0.36 ± 0.03	0.02	5.88	0.784	11.826	0.0202	S
	Group B	0.33 ± 0.04	0.40 ± 0.03	0.07	21.21	1.977	5.448	<0.0001	HS
	T-value	-0.866	3.652	-	-	-	-	-	-
	Cohen	0.316	1.33	-	-	-	-	-	-
	P-value	0.197 (NS)	0.0005 (HS)	-	-	-	-	-	-
Transverse abdominis TA	Group A	0.31 ± 0.05	0.34 ± 0.04	0.03	9.68	0.662	11.832	0.04	S
	Group B	0.29 ± 0.03	0.38 ± 0.02	0.02	31.03	3.529	3.738	<0.0001	HS
	T value	-1.328	3.464	-	-	-	-	-	-
	Cohen	-0.485	1.266	-	-	-	-	-	-
	P-value	0.097 (NS)	<0.001 (HS)	-	-	-	-	-	-
GMFM-88 sitting ability	Group A	47.03 ± 4.54	52.03 ± 4.64	5	10.63	1.09	3.742	0.0029	HS
	Group B	49.02 ± 5.24	58.58 ± 3.85	9.56	19.51	2.08	4.698	<0.001	HS
	T value	1.111	4.207	-	-	-	-	-	-
	Cohen	0.407	1.538	-	-	-	-	-	-
	P-value	0.137 (NS)	<0.001 (HS)	-	-	-	-	-	-

Before: pre-treatment; after: post-treatment; MD: mean difference; %: percentage of improvement; t value: paired t-test; T value: unpaired t-test; P-value: significance level at 0.05; S: significant difference (P<0.05); HS: highly significant (P<0.01); NS: non-significant difference (P>0.05); Cohen: Cohen's d effect size; mean ± SD: mean ± standard deviation; Sig.: significance

**Effect of WBV on sitting ability:** sitting ability, assessed using GMFM-88-dimension B, improved in both groups, with larger gains in group B (Table 2). Group A increased from 47.03 ± 4.54 to 52.03 ± 4.64 (10.63%, MD = 5, t = 3.742, P = 0.0029), while group B increased from 49.02 ± 5.24 to 58.58 ± 3.85 (19.51%, MD = 9.56, t = 4.698, P < 0.001). Post-treatment between-group comparison showed a significant difference favoring Group B (T = 4.207, P < 0.001), indicating enhanced postural control and functional sitting performance with WBV.

**Overall treatment effects:** both interventions led to significant improvements in abdominal muscle thickness and sitting ability. The WBV group consistently showed greater improvements across all parameters, suggesting that combining WBV with conventional physiotherapy provides superior effects on postural control, abdominal muscle development, and motor function in children with spastic hemiplegic cerebral palsy. Table 1 and Table 2 provide detailed demographic, baseline, and post-treatment values.

## Discussion

Our study investigated the influence of WBV therapy on the thickness and size of the abdominal muscles as well as sitting ability in spastic CP children over 12 weeks. The results of this study met our expected goals for enhancing abdominal muscle thickness in both groups. However, the study group demonstrated superior outcomes. Whole-body vibration (WBV) therapy showed benefits for participants' functional capacity, with a notable increase in gross motor function, abdominal muscle strength and thickness, and the sitting component of the GMFM-88. The improvements in sitting ability observed in this study were based on GMFM-88-dimension B, which specifically evaluates the postural control and functional stability from the sitting position. This analysis confirms the effectiveness of WBV in improving motor function and supporting improvements in posture and muscle mass. Overall, the observed outcomes provide robust evidence of the effectiveness of a 12-week WBV intervention in strengthening abdominal muscles and improving strength and posture in children with spastic hemiplegia.

The observed increases in abdominal muscle thickness following WBV intervention may be attributed to enhanced neuromuscular activation induced by vibratory stimuli. Whole-body vibration (WBV) stimulates muscle spindle activity and proprioceptive reflex pathways, leading to increased motor unit recruitment and tonic vibration reflex responses. These mechanisms promote repeated involuntary muscle contractions, which may facilitate muscle hypertrophy and improve neuromuscular coordination in children with cerebral palsy. Enhanced activation of deep stabilizing muscles, such as the transversus abdominis, likely contributed to improved trunk control and postural stability observed in the WBV group.

From a clinical perspective, the integration of WBV into conventional physiotherapy programs offers a time-efficient and engaging modality for improving core strength and sitting balance in children with spastic hemiplegic cerebral palsy. Improved trunk stability is fundamental for functional mobility, upper limb use, and participation in daily activities. The observed improvements in GMFM-88-dimension B suggest that WBV may enhance postural control in seated positions, which is a critical developmental milestone in pediatric neurorehabilitation.

Many studies supported the findings of our intervention. Studies based on the Bobath approach and other rehabilitation programs, including those by Mayston *et al*. and Ebert *et al*. suggested that a key factor contributing to improvement in motor function is the use of a comprehensive physical therapy program focusing on trunk strengthening and stimulation of the abdominal muscles through exercises performed in sitting, standing, and balance positions [28,29]. Additionally, Ali *et al*. used a WBV program similar to our study with side-to-side WBV in sitting and standing for 5 minutes each [30]. This intervention showed a significant effect on trunk and lower-limb strength, as well as on functional outcomes [30,31]. As for the frequency of WBV, using an intensity of 25-40 Hz for 10 minutes, 3 times a week over 3 months is effective in postural stability, trunk rotation, and motor performance [17]. This explains the superior outcomes observed in our study group over the control group.

While several studies have reported positive effects of WBV on muscle strength and functional performance in children with cerebral palsy, some studies have reported more modest benefits; for instance, Ruck *et al*. found no significant improvement in gross motor function following WBV in children with CP [32]. A systematic review by Saquetto *et al*. noted high methodological heterogeneity across WBV studies and classified the evidence quality as low to moderate [33]. These discrepancies may stem from variations in WBV parameters (frequency, amplitude, duration), participant characteristics (CP type, severity, age), or outcome measures. Our positive outcomes using a standardized 30 Hz protocol over 12 weeks suggest that specific, consistent parameters may be crucial for obtaining reliable benefits.

Increased muscle strength and abdominal muscle thickness with WBV were associated with improved motor function during a 4-week intervention [30]. In CP cases, motor function reduction is highly prevalent. This occurs because a cerebral lesion impairs the transmission of efferent signals from the motor cortex to muscle fibers, resulting in incomplete growth of muscles and bones [34-37]. This increase or decrease in thickness can be seen using an ultrasound, as muscle thickness varies in the resting state according to its strength [38]. Hussain *et al*. and others have found that WBV stimulates proprioceptive spinal circuits leading to the contraction of muscle fibers in the trunk and the lower limb and increasing thickness and power, reflecting on the children's motor performance [39-41]. Zanker *et al*. also established a strong correlation between muscle mass and strength [42]. All these findings are consistent with the results and methodology of our study. Additionally, Kantor *et al*.also demonstrated improvement in motor function and postural stability after a 3-month intervention of WBV therapy using 40 Hz intensity for 20 minutes, once a week [40]. Two experimental studies and a literature review also have supported the findings of our study, where WBV therapy has significantly improved functional ability and strength in children with spastic CP [43,44]. Lastly, the improvement observed in the sitting component of GMFM-88 (dimension B) after a 12-week intervention corresponds with the findings from Peungsuwan *et al*. where he showed WBV has improved both walking and standing, jumping and running balance (dimensions D and E in GMFM-88) compared to resistance training [17].

Whole-body vibration also induces mechanical forces on the body, which stimulate muscle growth. Myogenic and osteogenic stimuli are substantial during the WBV training, and they introduce muscle hypertrophy, contributing to an increase in bone mineral content and density [45-48].

To consolidate our findings, we can conclude that using WBV at a frequency of 30 Hz and 2 mm amplitude, combined with a regular physical therapy program, offers a preferable approach to enhancing the thickness and strength of the abdominal muscles. The absence of a sham WBV control group represents an important methodological limitation. Without a placebo-controlled condition, it is not possible to fully differentiate the specific physiological effects of WBV from potential placebo responses, increased participant engagement, or therapist-related performance bias. Children receiving WBV may have demonstrated greater motivation or effort due to the novelty of the intervention, which could have contributed to improved outcomes independent of the mechanical vibration stimulus. Future randomized controlled trials incorporating a sham WBV condition with similar positioning but no vibratory stimulus are necessary to better isolate the true therapeutic effects of WBV and strengthen causal inference.

**Limitations:** having a limited sample size of 30 children impacted the generalizability of the intervention to a broader spectrum. Additionally, the duration of the study was only 12 weeks, which limited the analysis of the long-term impact and the sustainability of our intervention. Moreover, there was also an absence of a sham WBV group, which posed a challenge in isolating the specific effects of WBV from potential placebo effects. We recommend, for future research, investigating WBV therapy combined with regular physical therapy with a broader sample and a prolonged follow-up period, as well as more rigorous control measures to warrant further validation and improvement and extend the implications of the current findings. Although the sample size was calculated based on a large pilot effect size, such estimates may overstate the true intervention effect and consequently overestimate statistical power. Therefore, the present study may be underpowered to detect smaller but clinically meaningful differences, particularly for secondary outcomes. Future studies with larger sample sizes are recommended to confirm these findings and reduce the risk of type II error. The use of multiple statistical comparisons without formal correction represents a limitation of the present study and may increase the risk of false-positive findings. Nevertheless, this approach was chosen to preserve statistical power in a relatively small sample and to explore potential therapeutic effects across several relevant muscle groups. Future studies with larger sample sizes should apply appropriate correction methods to confirm these findings.

**Recommendation:** to advance the understanding of WBV and its application in children with spastic hemiplegic CP, certain recommendations have been proposed: 1) longitudinal studies with a focus on extended interventional periods are recommended to observe the cumulative benefits over extended periods; 2) investigating the dose-response relationship of WBV and exploring the variations in frequency, amplitude, and duration during the intervention; 3) including a sham WBV control group to differentiate between the specific effects of WBV and potential placebo effects; 4) improve adherence measurement monitoring for the WBV intervention protocols; 5) improving and expanding the functional outcome assessment beyond sitting ability for comprehensive evaluation; 6) incorporation of electromyography and biomechanical assessments to quantify muscle activation and function as objective measures; 7) age groups' specific considerations and potential modifications to the WBV protocol for different age groups within the pediatric population. These recommendations seek to improve the utilization of WBV as a treatment, offering a detailed comprehension of its advantages and factors to consider for enhancing therapeutic results in children with spastic hemiplegic CP.

## Conclusion

The current investigation revealed a notable enhancement in the thickness of the core muscles and sitting balance in both the experimental and the comparison cohorts. These advancements stemmed from core strength using exercise physical therapy alongside WBV, contributing to the augmentation of postural control and motor function as well.

***What is known about this topic***

- *Children with spastic hemiplegic CP often have weak abdominal muscles and poor trunk control, affecting posture and functional mobility;*
- *Whole-body vibration can enhance muscle strength, bone mineral density, and postural stability in children with cerebral palsy;*
- *Ultrasonography is a reliable method to measure abdominal muscle thickness and assess training effects in this population*.

***What this study adds***

- *Whole-body vibration combined with conventional therapy significantly increases abdominal muscle thickness compared to therapy alone in spastic hemiplegic children;*
- *Whole-body vibration leads to greater improvements in sitting ability, as measured by GMFM-88, than conventional therapy alone;*
- *A 12-week whole-body vibration program at 30 Hz and 2 mm amplitude is effective for enhancing trunk muscle strength and functional sitting balance in this group*.
